# Bridging the Gap: Improving Health Outcomes for Adults With Intellectual and Developmental Disabilities

**DOI:** 10.15766/mep_2374-8265.11630

**Published:** 2026-08-21

**Authors:** Nicole Reynoso-Vasquez, Kathryn Davis, Gregory Sabel, John Paul Sánchez

**Affiliations:** 1 Assistant Professor, Department of Medicine, Rutgers New Jersey Medical School; 2 Resident, Department of Medicine, Rutgers New Jersey Medical School; 3 Medical Student, New York Medical College School of Medicine; 4 Executive Director, Building the Next Generation of Academic Physicians (BNGAP)

**Keywords:** Disability, Developmental Delay, Developmental Disability, Intellectual Disability, Medically Complex Patients, Health Disparities, Preventative Care, Health Promotion, Primary Care

## Abstract

**Introduction:**

Individuals with intellectual and developmental disabilities (IDD) are often affected by multisystem diseases and are considered medically complex. Due to their high need for care coordination, these patients frequently experience health disparities that lead to higher morbidity. A comprehensive primary care approach can lead to improved health outcomes through health promotion and disease prevention.

**Methods:**

We implemented a 60-minute interactive workshop for medical students, internal medicine and pediatric residents, and faculty physicians to increase familiarity with the care of adult patients with IDD and complex medical needs. The workshop consisted of a PowerPoint presentation that included interactive polling and an interactive case study. The workshop was implemented at 2 professional conferences and at the Rutgers New Jersey Medical School Medical Education Conference.

**Results:**

There was a total of 30 participants. Comparison of responses to pre- and postworkshop questionnaires showed a statistically significant (*P* < .001) improvement in participants’ confidence regarding their ability to define disability, familiarity with the International Classification of Functioning, Disability, and Health (ICF) model, knowing what entails medical complexity, caring for patients with IDD and complex medical needs, and familiarity with health disparities experienced by patients with IDD. Participants valued the workshop's interactive and engaging nature, particularly the case discussion and application of the ICF model.

**Discussion:**

This workshop offered an interactive and effective method for increasing trainees’ and faculty's knowledge of caring for patients with IDD, reinforcing the importance of comprehensive and longitudinal primary care assessments to minimize health disparities in this patient population.

## Educational Objectives

By the end of this activity, learners will be able to:

1. Differentiate between developmental delays and intellectual and developmental disabilities (IDD).
2. Understand disability through the International Classification of Functioning, Disability, and Health model and use it as a framework to guide patient care.
3. Describe factors that contribute to medical complexity.
4. Outline a comprehensive primary care assessment for adult patients with IDD.
5. Examine the health disparities faced by patients with IDD.

## Introduction

Adults with intellectual and developmental disabilities (IDD) are usually considered to be medically complex, and tend to be affected by multisystem diseases that require specialty care, have recurring presentations, and are highly dependent on care coordination.^1^ These conditions include but are not limited to cerebral palsy, trisomy 21, and autism spectrum disorders. Although rewarding, caring for patients with medical complexity can be both challenging and intimidating. Often, physicians find that they have not received comprehensive training and education in caring for this special population until they reach residency.^2^

Due to their medical complexity, these patients often face increased risks of fragmented care and sentinel events, and are vulnerable to experiencing health service inequities.^3^ Barriers to health service access result in ineffective monitoring of preventable conditions and missed opportunities to promote health and well-being.^4^ Consequently, these patients are more likely to visit the emergency department and be hospitalized for conditions that could be managed through outpatient services.^1,3^

Despite the great need for medical education and proficiency in the care of patients with IDD, many physicians and other health care professionals lack sufficient training in caring for this patient population.^5^ As the number of pediatric patients with medical complexities who reach adulthood increases, it is imperative to expose medical students and residents to the care of this patient population early in their training to gain familiarity and competence in this area.^6^ Because these patients often require multidisciplinary and multispecialty care, doctors across all specialties should have a level of comfort with caring for patients with complex medical needs.^2^

Despite the known health disparities faced by individuals with IDD, standardized education in disability care remains inconsistently integrated across the medical training spectrum. Current modules published on *MedEdPORTAL* have successfully introduced disability health to preclinical medical students using didactic panels and interactive video sessions.^7,8^ Recent scholarship has further expanded this undergraduate focus to include the study of ableism,^9^ shared decision-making,^10^ and interprofessional communication strategies specifically for the IDD population.^11^

However, a significant gap remains in providing clinical frameworks for postgraduate learners and practicing clinicians in the adult sector. While robust curricula exist for pediatric residents regarding medical complexity^12^ and transition to adulthood,^13,14^ “minimal provider training” remains a primary barrier to successful care coordination in adult medicine.^13^ Our workshop addresses this identified training gap by providing an International Classification of Functioning, Disability, and Health (ICF)-based clinical reasoning framework for third- and fourth-year medical students, residents, and practicing faculty to manage the longitudinal care of adults with IDD.

## Methods

### Participants

This 60-minute workshop was designed for medical students (years 3 and 4), residents across all specialties, and faculty physicians. While the content was shared with internal medicine and pediatric residents, the module is intended for clinicians at any training level because patients with IDD and medical complexity require multidisciplinary care, making it inevitable that physicians across all disciplines will encounter them.

### Procedure

The session was led by the co-authors, who are specialists in internal medicine and pediatrics with experience in caring for adult and pediatric patients in both inpatient and outpatient settings. This workshop was developed to gain a deeper understanding of what it means to have IDD, as well as medical complexity, and to develop proficiency in approaches to their care in the primary care setting. This workshop introduced the ICF model as a tool for conducting a baseline assessment of patients with disabilities and for developing a patient-centered approach to care coordination. This workshop also discussed the health disparities present in this patient population, including limited access to primary care and, therefore, preventative care, leading to a risk for increased morbidity.

A PowerPoint presentation was created to present this workshop. Interactive polling and a case presentation were used during the lecture to facilitate an interactive discussion. Additionally, educational materials were provided in a separate handout outlining best practices for caring for adult patients with IDD and complex medical needs in the primary care setting.

### Implementation

The first implementation of this module was a virtual presentation at the Building the Next Generation of Academic Physicians (BNGAP) Medical Education Conference. It was presented to a group of health care professionals ranging from medical students to faculty members at various medical institutions. The second implementation was an in-person presentation at the Rutgers New Jersey Medical School Medical Education Conference for internal medicine and pediatric residents. Finally, the third implementation was a virtual presentation at the BNGAP Academic Writing Fellowship Orientation, presented to health care professionals at various training and faculty levels. Surveys were distributed before and after the workshop to assess baseline familiarity with the lecture content and confidence in caring for this patient population, as well as to evaluate the proficiency gained following the seminar.

The module began with the distribution of preworkshop evaluation forms (Appendix A). After this, a PowerPoint slideshow (Appendix B) was presented, which included multiple interactive activities, such as polling and a detailed case discussion during the presentation. The interactive activities allowed participants to apply the ICF model, which was introduced during the presentation, and participate in a case discussion, which highlighted the importance of care coordination in patients with complex medical needs and IDD. After the slide presentation, postevaluation surveys (Appendix C) were distributed to assess the knowledge gained during the workshop and gather feedback on methods to improve it.

The workshop's content was revised and updated on subsequent presentations based on the results and feedback from earlier sessions. Following the first implementation, a detailed case discussion was added to demonstrate how to approach a patient with IDD and medical complexity in the primary care setting and apply the ICF model to their care. In addition, a teaching guide (Appendix D) was developed following the third session, providing tools and best practices for the care of patients with IDD with complex medical needs. This form provides additional information on how to utilize the ICF model in a clinical setting and how to incorporate it into a patient's note during a clinic encounter. Additional resources on care transitions and guardianship are also provided.

The workshop can be delivered in 60–75 minutes with appropriate modifications to time. A facilitator guide (Appendix E) was created to provide additional details and guidance to future facilitators. This workshop could be delivered by 1 or 2 facilitators who, ideally, would have experience working with patients, either pediatric or adults, with IDD. Before the session, speakers are required to dedicate at least 3 hours to reviewing the module (see Appendix B), as well as the facilitator guide (see Appendix E), which serves as a step-by-step guide through the content. To optimally deliver the module, the following items should be provided: audiovisual equipment for the PowerPoint display; tables/desks and chairs arranged for small- to medium-sized group discussions; printed copies of the best practices handout form (see Appendix D); and evaluation forms (see Appendices A and C). The module could also be delivered in a virtual format and modified to the convenience of facilitators.

### Suggested Agenda and Timeline

• Preworkshop evaluation: 2–3 minutes
• Slides 1–8, objectives, presentation introduction, and description of the ICF model: 5 minutes
• Slides 9–13, statistics with interactive group activity: 2 minutes
• Slides 14–20, definition of developmental delay, global developmental delay, and IDD with interactive group activities: 20 minutes
• Slides 21–35, description of medical complexity, approach to the patient, case discussion in large-group format, and definition of guardianship: 20 minutes
• Slides 36–39, health disparities, health promotion, and summary: 10 minutes
• Postworkshop evaluation: 2–3 minutes

### Data Analysis

The pre- and postevaluation surveys (see Appendices A and C) were developed using Qualtrics and incorporated a Likert scale to measure participants’ self-reported confidence in caring for patients with IDD and medical complexities. These surveys also aimed to assess the effectiveness of the workshop in meeting its stated educational objectives. Additionally, the pre-evaluation survey elicited participants’ demographic information. The postevaluation survey included questions on what they liked and what they would improve in the workshop. Quantitative data from the pre- and postworkshop surveys was analyzed using SPSS Version 23. We used the Wilcoxon matched-pairs signed-rank test to measure the difference between 2 dependent (paired) samples and assess whether there is a significant difference in their pre- and postworkshop medians. We did not conduct a formal analysis of the qualitative data because of a limited number and breadth of responses but rather share illustrative quotes.

## Results

The workshop was implemented 3 times. Thirty participants completed a presession evaluation, including 6 who identified as medical students, 14 as residents, 9 as faculty/physicians (MD, DO, NP, PA, PhD, other), and 1 as other. In terms of race/ethnicity, 8 participants identified as LHS+ (Latina, Latino, Latinx, Latine, Hispanic, or of Spanish Origin+), 4 as Black/African American, 9 as White, 11 as Asian, and 1 as Middle Eastern. Nineteen identified as women and 10 as men. Twenty-two identified as straight/heterosexual, and 8 as lesbian, gay, bisexual, and transgender (LGBT).

A total of 26 participants completed the pre- and postworkshop evaluation. Of these participants, 6 identified as medical students, 13 as medical residents or fellows, 4 as academic faculty, 2 as physicians in nonacademic practice, and 1 as “other professional role.” For the statement “I can define what it means to have a disability,” the preworkshop median was 4 and the postworkshop median was 5, *P* < .001; for “I am familiar with the ICF model and I feel comfortable applying it in a clinical setting,” the preworkshop median was 1 and the postworkshop median was 4, *P* < .001; for “I know the difference between developmental delay, developmental disability and intellectual disability,” the preworkshop median was 3 and the postworkshop median was 5, *P* < .001; for “I know what entails medical complexity,” the preworkshop median was 4 and the postworkshop median was 5, *P* < .001; for “I feel confident caring for patients with developmental/intellectual disability and complex medical needs,” the preworkshop median was 2 and the postworkshop median was 4, *P* < .001; and for “I am familiar with some of the health disparities faced by patients with IDD,” the preworkshop median was 2 and the postworkshop was median was 4, *P* < .001.

The utility of the session, as measured by the degree of agreement with the statement in Appendix C, “This lecture has changed my approach to the care of disabled and medically complex patients,” appears to have been useful across all professional roles. From the participants who completed both pre- and postworkshop evaluations, 25 of the 26 rated the statement. Four of the medical student participants strongly agreed and 2 somewhat agreed with the statement. Nine of the medical residents or fellows strongly agreed, whereas 3 somewhat agreed. From the academic faculty, 2 strongly agreed and 2 somewhat agreed. Both nonacademic physicians and “other professional role” respondents strongly agreed with the statement. Respondents complimented several aspects of the workshop, describing it as “interactive” and “engaging.” Four participants appreciated the case discussion of Kira and valued the application of the ICF model to the case. One respondent wrote, “Case example was effective with segments conveying different dilemmas and challenges.” Three respondents noted the clear definitions and the use of images to highlight preventive health measures and the ICF model.

In terms of ways to improve the workshop, 1 respondent suggested adding more specific preventive measures by disability category: “I liked the specifics such as that wheelchair dependent people might need earlier osteoporosis screening and would like more specifics like that.” Another individual recommended providing specific resources for patients with IDD in New Jersey, the state where this workshop was delivered. In the future, other versions of this workshop could incorporate state-specific resources tailored to the location of the learners, making the workshop more broadly applicable.

## Discussion

While previous works have addressed various concerns regarding the inclusion of individuals with IDD,^1–4^ this workshop addresses a critical gap in the current educational material available. By focusing on the unique role of primary care in addressing inequalities and inadequacies in health care for adults with IDD, awareness of disparities can be acknowledged, addressed, and ultimately eliminated.^9^ While prior educational resources have emphasized pediatric populations or general disability awareness,^3^ this workshop's unique perspective allows for a more holistic and realistic view of the challenges faced by this population. By improving clinician confidence and knowledge, this workshop addresses the critical training gap where physicians often do not receive comprehensive education on this population until residency.^1^ Early education that promotes proactive quality management in outpatient settings may help reduce emergency department visits and hospitalizations.^2,3^

One strength of this innovative module was the use of interactive elements, which included case-based discussions and application of the ICF model. These activities expand on the traditional lecture format, enabling learners of all levels to practice clinical reasoning in real-world scenarios while reflecting on system-level barriers faced by adults with IDD. Survey responses indicated that these interactive sections were highly valued by participants, who appreciated the opportunity to apply the theoretical frameworks they had learned to a detailed case. This exercise also serves as an active learning tool, strengthening the lessons learned when teaching about complex, multidisciplinary care.

The flexibility of the format and audience is a strength of this module, as it can be implemented both virtually and in person, and it has reached a broader audience of medical students, residents, and faculty. This adaptability is paramount to generalizability and utility across varied institutions, and resources such as the best practices handout (see Appendix A) allow for reinforcement and implementation of concepts in clinical practice. While our evaluation demonstrated a statistically significant increase in participants’ immediate confidence and knowledge regarding the ICF model and medical complexity (*P* < .001), we view these as the necessary first steps toward a broader anticipated impact on clinical practice. Resources like the best practices handout are designed to provide the “how-to” framework for clinical implementation. This description of the handout's utility reflects our intended goals for the resource to serve as a tool for reinforcement rather than a measured outcome, as our evaluation did not directly assess longitudinal changes in clinical behavior or patient outcomes.

Despite these strengths, several limitations must be acknowledged. First, the workshop was delivered to relatively small groups at academic conferences, which may limit generalizability to other settings such as community-based training programs or nonacademic institutions. Second, the evaluation primarily relied on self-reported confidence and knowledge measures, which, while valuable, do not directly assess changes in clinical behavior or patient outcomes. Future studies could incorporate longitudinal assessments to determine whether the workshop leads to sustained improvements in practice. Additionally, for the statement “This lecture has changed my approach to the care of disabled and medically complex patients” in the postsurvey, there were too few responses to statistically compare differences by learner level. Finally, some participants requested more granular, condition-specific guidance on preventive health measures and state-specific resources, highlighting an opportunity to explore this topic with additional modules in the future.

Overall, the high level of agreement across all groups regarding the “change in approach” to clinical practice following this workshop suggests that the workshop successfully bridged the knowledge gap for learners at multiple stages of medical training and practice. Looking forward to other areas in need of attention, expansion of this workshop could include the development of additional modules that address specific specialty or subspecialty perspectives, interprofessional collaboration, or community partnerships with local organizations. Workshops embedded into medical school or residency curricula may strengthen early exposure and normalization of training on the care of adults with IDD. Given the persistent and problematic health disparities faced by adults with IDD, curricula that prepare physicians to deliver equitable and patient-centered care are essential.^7,8^

## Appendices


Presession Evaluation.docxBridging the Gap.pptxPostsession Evaluation.docxBest Practices Handout.docxFacilitator Guide.docx
*All appendices are peer reviewed as integral parts of the Original Publication.*
