# Supplementary material for: Bridging the Gap: Improving Health Outcomes for Adults With Intellectual and Developmental Disabilities
Source: MedEdPORTAL. 2026 Aug 21;22:11630. doi: 10.15766/mep_2374-8265.11630 (PMC13493612; doi:10.15766/mep_2374-8265.11630)
Supplement: Supplementary file 1 [file mep_2374-8265.11630-s001.zip › C. Postsession Evaluation.docx]

**Writing fellowship Post**

**This is the post workshop survey. This document should be distributed to the participants after the workshop.**

**Start of Block: Demographics**

Q1 Please enter the 5-digit PIN you created using the FIRST initial of your LAST NAME and the last 4 digits of your phone number. (ie: P1914 you will also be putting this on your post survey to link them)

________________________________________________________________

**End of Block: Demographics**

**Start of Block: Care of the complex patient**

Q2 Please rate how much you agree with the following statements

|  | Strongly disagree (1) | Somewhat disagree (2) | Neither agree nor disagree (3) | Somewhat agree (4) | Strongly agree (5) |
| --- | --- | --- | --- | --- | --- |
| I can define what it means to have a disability |  |  |  |  |  |
| I am familiar with the ICF model and I feel comfortable applying it in a clinical setting |  |  |  |  |  |
| I know the difference between developmental delay, developmental disability and intellectual disability |  |  |  |  |  |
| I know what entails medical complexity |  |  |  |  |  |
| I feel confident caring for patients with developmental/intellectual disability and complex medical needs |  |  |  |  |  |
| I am familiar with some of the health disparities faced by patients with IDD |  |  |  |  |  |
| This lecture has changed my approach to the care of disabled and medically complex patients |  |  |  |  |  |

**End of Block: Care of the complex patient**

**Start of Block: Feedback**

Q3 What did you like about this workshop?

________________________________________________________________

________________________________________________________________

________________________________________________________________

________________________________________________________________

________________________________________________________________

Q4 What would you improve about this workshop?

________________________________________________________________

________________________________________________________________

________________________________________________________________

________________________________________________________________

________________________________________________________________

**End of Block: Feedback**
