# Supplementary material for: Bridging the Gap: Improving Health Outcomes for Adults With Intellectual and Developmental Disabilities
Source: MedEdPORTAL. 2026 Aug 21;22:11630. doi: 10.15766/mep_2374-8265.11630 (PMC13493612; doi:10.15766/mep_2374-8265.11630)
Supplement: Supplementary file 1 [file mep_2374-8265.11630-s001.zip › D. Best Practices Handout.docx]

**Tools and best practices in primary care for the care of patients with intellectual and developmental disabilities with complex medical needs**

**This document can be used as supplemental material for the facilitator(s) before the workshop and to the participants after the workshop to familiarize themselves with available resources that can be integrated into their practice.**

1) **The role of the ICF model in primary care practices**

Patient-centered approach that allows the provider to identify and prioritize aspects of functioning that are most crucial to address, aligning them with the patient’s goals and individual needs

a) The ICF checklist is an optional resource that can be used by healthcare providers with the patient and their caregiver during a dedicated visit for this assessment. Baseline assessment recommended for new patients and subsequently depending on interventions to track progress. This assessment can be part of the patient’s HPI and guide their plan of care.
   i) “This is a checklist of major categories of the International Classification of Functioning, Disability and Health (ICF) of the World Health Organization. The ICF Checklist is a practical tool to elicit and record information on the functioning and disability of an individual. This information can be summarized for case records (for example, in clinical practice or social work). The checklist should be used along with the ICF or ICF Pocket version” World Health Organization. *ICF Checklist: Version 2.1a, Clinician Form for the International Classification of Functioning, Disability and Health.* Geneva, Switzerland: World Health Organization; 2003. Accessed December 16, 2025. [https://www.who.int/publications/m/item/icf-checklist](https://www.who.int/publications/m/item/icf-checklist?utm_source=chatgpt.com)
   ii) For additional information on the ICF Checklist, the following optional resource provides access to the checklist, which is a short-form application derived from the full ICF and intended for use in clinical practice, surveys, and research. The checklist is organized according to the primary ICF components.
       (1) World Health Organization. ICF Checklist: Version 2.1a, Clinician Form. Published September 1, 2003. Accessed December 16, 2025. [https://www.who.int/publications/m/item/icf-checklist](https://www.who.int/publications/m/item/icf-checklist?utm_source=chatgpt.com)
   iii) Consider creating smartphrases depending on the institutional EMR system to record this information electronically.

2) **Begin conversations regarding transitions from pediatric to adult care early.**
   a) Refer to the American Academy of Pediatrics for best practices regarding transition of care and tools for transition readiness, access to these links is optional and provided for additional reference.
      i) White PH, Cooley WC; Transitions Clinical Report Authoring Group; American Academy of Pediatrics; American Academy of Family Physicians; American College of Physicians. Supporting the health care transition from adolescence to adulthood in the medical home. *Pediatrics.* 2018;142(5):e20182587. doi:10.1542/peds.2018-2587. <https://publications.aap.org/pediatrics/article/142/5/e20182587/38577/Supporting-the-Health-Care-Transition-From>
         (1) Description: this link provides the clinical report from the American Academy of Pediatrics providing guidance on the transition of adolescents and young adults from pediatric to adult care. It outlines strategies within the medical home to promote successful health care transitions.
      ii) Got Transition®. The six core elements of health care transition™. Got Transition/Center for Health Care Transition Improvement. Accessed December 16, 2025. [https://www.gottransition.org/six-core-elements/](https://www.gottransition.org/six-core-elements/?utm_source=chatgpt.com)
          (1) Description: this link provides an overview of the Six Core Elements of Health Care Transition, a framework to guide youth in transitioning from pediatric to adult-centered care, and provides resources to support clinical practices in implementing effective health care transitions.
3) **Guardianship should be addressed for patients transitioning to adult care beginning at age 17.**
   a) Visit your state’s court website for additional information and resources for patients
   b) The following links from the New Jersey and New York Judiciary provide information about guardianship and resources for families and individuals navigating this process in these states. Access to these links is optional and provided for additional reference.
      i) New Jersey Courts. Guardianship. New Jersey Judiciary. Accessed December 16, 2025. <https://www.njcourts.gov/courts/civil/guardianship>
      ii) New York State Courts. Guardianship 17A. New York State Unified Court System. Accessed December 16, 2025. <https://www.nycourts.gov/courthelp/guardianship/17A.shtml>
