# Supplementary material for: Bridging the Gap: Improving Health Outcomes for Adults With Intellectual and Developmental Disabilities
Source: MedEdPORTAL. 2026 Aug 21;22:11630. doi: 10.15766/mep_2374-8265.11630 (PMC13493612; doi:10.15766/mep_2374-8265.11630)
Supplement: Supplementary file 1 [file mep_2374-8265.11630-s001.zip › E. Facilitator Guide.docx]

**Facilitator Guide**

**The facilitator guide is to be used throughout the workshop as a guide to the facilitator(s) as they present the module. It is recommended that the facilitator(s) review this document before the workshop to familiarize themselves with the content and flow of the presentation.**

**Overall Goals**

**Objectives**

- Differentiate between developmental delays and intellectual and developmental disabilities.
- Understand disability through the international classification of Functioning (ICF)-model and use it as a framework to guide patient care
- Describe factors that contribute to medical complexity
- Outline a comprehensive primary care assessment for adult patients with IDD
- Examine the health disparities faced by patients with IDD

**Suggested Agenda and Timeline**

- Pre-workshop evaluation: 2–3 minutes
- Slides 1–8, including objectives, introduction and introduction to the ICF model: 5 minutes
- Slides 9–13, Statistics with interactive group activity: 2 minutes
- Slides 14–20, definition of developmental delay, global developmental delay and IDD with interactive group activities : 20 minutes
- Slide 21–35, description of medical complexity, approach to the patient, case discussion in large group format, definition of guardianship: 20 minutes
- Slide 36-39, Health disparities, health promotion, and summary 10 minutes
- Postworkshop evaluation: 2–3 minutes

**Handouts and Materials**

- Computer with audio capability and connection to projector
- Internet
- Facilitator Instructional Guide
- Pre-evaluation form
- Post-evaluation form
- PowerPoint presentation
- Case scenarios

**Slide Instructions**

**Slide 1:** Title page

*The facilitator(s) should introduce themselves to the audience.*

**Slide 2:** Objectives

*The facilitator(s) will present the objectives for the educational intervention.*

**Slide 3:** Introduction

*The facilitator(s) will present the introduction with the following additional information.*

- Caring for patients with IDD and medical complexity is rewarding but can be challenging, especially as many physicians lack adequate training until residency and often feel unprepared in both inpatient and primary care settings.
- As more pediatric IDD patients transition to adulthood and the aging population grows, early exposure during medical training is critical to build competence and meet rising healthcare demands.
- Regardless of specialty, physicians must be prepared for the multisystem, evolving needs of this population, which requires coordinated, multidisciplinary care to avoid fragmented services and poor outcomes.
- Barriers to access often lead to missed preventive care and higher rates of emergency visits and hospitalizations for otherwise manageable conditions.
- Addressing care gaps and understanding these patients' unique needs is essential to reducing health disparities.

**Slide 4:** Disability definition

*The facilitator(s) will define “disability” according to the definition provided by the WHO*

The term "disability" includes a diverse group of people with a wide range of needs. According to the World Health Organization disability has three dimensions: impairment, activity limitation and participation restrictions. It is an umbrella term that is used to describe any condition of the body or mind that makes it difficulty for the person with the condition to do certain activities and as a result interact with the world around them.

**Slide 5:** Disability and the WHO-ICF Model

*The facilitator(s) will define disability according to the WHO-ICF model*

- The world health organization further described disability by creating the international classification of functioning, disability, and health, or the ICF model, which was approved for use by the WHO in 2001, and further classified and published the model for children and young adults in 2007.
- The components of the ICF model, contain a lot of the concepts that are part of the definition for disability provided by the WHO. Overall, in the ICF model, functioning and disability are multi-dimensional concepts relating to body functions and structures and their impairments, activities and activity limitation, participation and restrictions in the individual’s life.
- However, compared to this prior definition of disability, this model looks at the role of the patient’s environment, associated health conditions, the interaction between them, and how these factors affect that person's quality of life, rather than focusing solely on the disability itself, looking at environmental and personal factors that affect someone’s life experience as either facilitative or a barrier.

***Additional background information for the facilitator to better understand the ICF model:***

Understanding the ICF Framework

The Biopsychosocial Shift: The ICF represents a shift from a "medical model" (which focuses on the diagnosis) to a "biopsychosocial model". It treats "functioning" and "disability" as a dynamic interaction between a person’s health condition and their environment.

Core Domain Definitions:

Body Functions & Structures: These are the physiological functions (e.g., joint function, cognitive processing) and anatomical parts (e.g., limbs, organs).

Activities: This refers to the execution of a task by an individual (e.g., walking, communicating).

Participation: This is the involvement in a life situation or the world around them (e.g., attending school, having a job, community life).

The Contextual Factors (The "Interplay"):

Environmental Factors: These are external to the individual and can be facilitators (e.g., ramps, supportive family) or barriers (e.g., negative attitudes, lack of insurance).

Personal Factors: These are internal to the individual, such as age, gender, education, and motivation, which influence how disability is experienced.

The CDC provides a qualifier scale (0–4) to measure the extent of a problem within each domain, ranging from "no problem" to "complete problem". This allows clinicians to move beyond a binary "disabled vs. not disabled" view to a nuanced assessment of functional needs.

**Slide 6:** The ICF model

*The facilitator(s) will further describe the ICF model as a framework*

- Framework for describing and organizing information on functioning and disability.
- The aims of the ICF include:
- Establishing a common language for describing health (and disability) and health-related states to improve communication between different healthcare workers, researchers, policy makers, and the public
- The ICF integrates the major models of disability and recognizes the role of the environmental factors in the creation of disability, as well as the relevance of associated health conditions and their effects

**Slide 7:** Models of disability integrated in the ICF framework

*The facilitator(s) will describe existing models of disability and explain which models are integrated into the ICF framework*

- The models of disability emphasize that how people think about disability, affects how they feel about disability. Although people have various individual perspectives on disability, these viewpoints can be categorized into three overarching models of disability—moral, medical, and social. Each model addresses the perceived causes of disability, appropriate responses, and deeper meanings.
- The ICF model integrates the medical and social models. The medical model focuses on biological impairments while the social model focus on the societal and environmental factors that affect an individual’s experience.
- The medical model views disability as a feature of the person that is directly caused by a disease or health condition. It focuses on the "Body Functions and Structures" and "Impairments". While the social model views disability as a socially created problem rather than an attribute of the individual. It emphasizes how "Environmental Factors" can act as barriers to a person's full "Participation" in society

**Slide 8:** The ICF model

*The facilitator(s) will further explain the components of the ICF model and how to use it in the clinical setting*

- Now looking at the model itself, this is the graphic released by the CDC/WHO and how we can use and apply the model. If you go on the CDC website they provide instructions on how to use this and examples of the components in each domain. They also have a qualifier scale from zero being no problem at all to 4 being a complete problem vs not applicable or not specified.
- Starting at the top looking at the health condition in order to better understand the context of the disability. And then moving down to body functions and structures, how these impairments can affect activity and how this activity limitation can lead to participation restriction with the world around them. Now looking down at the diagram, we can see how environmental factors and personal factors can further affect these same three things.
- The diagram itself represents this balance. The top half of the diagram (Health Condition/Body Structures) represents the **Medical perspective**. The bottom half (Environmental Factors) and the "Participation" wing represent the **Social perspective**.

**Slide 9:** True or False interactive activity

The facilitator(s) will engage the audience in a true or false question allowing the participants 1-2 minutes to answer the question. The actual statistic and answer will be revealed on the next slide.

- Let’s start out with a quiz. True or false, fewer than 10% of adults in the Unites States have a disability

Use an interactive program such as SLIDO.com or Polleverywhere.com to allow anonymous audience participation. If you don’t have access to such programs, you can have individuals raise their hands in response to the question.

**Slide 10:** Statistics from the CDC

*The facilitator(s) will present statistical data from the CDC*

- As of 2024 according to the CDC, more than 1 in 4 adults (28.7 percent) in the United States have some type of disability.

**Slide 11:** Statistics *(cont.)*

*The facilitator(s) will present statistical data from the CDC*

- 13.9 percent of U.S. adults have a cognition disability with serious difficulty concentrating, remembering, or making decisions.
- 12.2 percent of U.S. adults have a mobility disability with serious difficulty walking or climbing stairs.
- 7.7 percent of U.S. adults have an independent living disability with difficulty doing errands alone.
- 6.2 percent of U.S. adults are deaf or have serious difficulty hearing
- 5.5 percent of U.S. adults have a vision disability with blindness or serious difficulty seeing even when wearing glasses.
- 3.6 percent of U.S. adults have a self-care disability with difficulty dressing or bathing.

**Slide 12:** Statistics *(cont.)*

*The facilitator(s) will present statistical data from the CDC*

Adults with disabilities are more likely to be obese, smoke, have heart disease, and diabetes:

- 40.5 percent of adults with a disability have obesity while 30.3 percent of adults without a disability have obesity.
- 20.9 percent of adults with a disability smoke while 10.2 percent of adults without a disability smoke.
- 10.4 percent of adults with a disability have heart disease while 3.7 percent of adults without a disability have heart disease.
- 16.6 percent of adults with a disability have diabetes while 7.9 percent of adults without a disability have diabetes.

**Slide 13:** Statistics *(cont.)*

*The facilitator(s) will present statistical data from the CDC*

Healthcare access barriers for working-age adults include

- 1 in 4 adults with disabilities 18 to 44 years do not have a usual healthcare provider
- 1 in 4 adults with disabilities 18 to 44 years have an unmet healthcare need because of cost in the past year
- 1 in 6 adults with disabilities 45 to 64 years did not have a routine check-up in the past year

This data highlights the diverse needs, challenges, and barriers faced by individuals with IDD. These issues may stem from the nature of their disability, but are often worsened by gaps in personal, environmental, and systemic support. This raises important questions: Why do these disparities persist, and how can we effectively address them?

**Slide 14:** Examples of how disability can present

*The facilitator can read the text off the slide as written below and can provide their own examples of the various ways a disability can present or develop*

Disability can be:

- Related to conditions that are present at birth and may affect functions later in life including cognition, mobility, vision, hearing, behavior and other areas
- It can be associated with developmental conditions that become apparent during childhood
- It can be Acquired
- Related to injury such as a Traumatic brain injury or spinal cord injury
- Associated with longstanding conditions such as complications developed in patients with diabetes (vision impairment, amputations)
- It can also be progressive, static, or intermittent think of a patient with multiple sclerosis

**Slide 15:** Definitions: developmental delay, global developmental delay and IDD

*The facilitator(s) will define the above terms*

- When thinking about intellectual or developmental disabilities, there are further distinctions that are important to recognize. A lot of times these are terms that we use interchangeably in practice however they are not quite the same.
- A **developmental delay** means a child **is not meeting expected milestones** and is continually behind in gaining skills expected by a certain age. It may affect one area (e.g., motor or speech) or **multiple areas**, in which case it's called **global developmental delay**.
- Some delays are temporary and improve with early intervention, while others may signal a long-term condition or developmental disability. Early identification is key to preventing poor outcomes and allows for screening of related health issues.
- After age 5, cognitive and adaptive functioning can be assessed to diagnose intellectual disability (ID).
- We can diagnose someone with **intellectual disability** after 5 years old because we can now test for cognition and adaptive behavior- but what does this mean? An intellectual disability is a lifelong condition that manifest during the developmental years, characterized by significant limitations in both intellectual functioning (cognition), and adaptive behaviors that generally include social and other everyday skills.
- The diagnosis is made by a specialist either in development or neurologist with validated tools that test both cognition and adaptive behavior. Intellectual disability can be further classified depending on the IQ score, which many of us may be familiar with.
- **Developmental disability** then, can be inclusive of intellectual disability; and typically used with individuals who:
  - Have a chronic disability that is due to mental and/or physical impairment
  - Are diagnosed with the disability before 22 years old, with the disability emerging during their developmental period and lasting throughout their lifetime
  - Have substantial functional limitations in their activities of daily living such as moving, learning, and communicating

**Slide 16:** Interactive activity, common causes of IDD

*The facilitator will take 1-2 minutes to engage in a discussion of common causes of IDD*

Use an interactive program such as SLIDO.com or Polleverywhere.com to allow anonymous audience participation. If you don’t have access to such programs, you can have individuals raise their hands in response to the question.

- What are some common causes of IDD?

**Slide 17:** Examples of common causes of IDD

*The facilitator(s) will read the listed examples of causes of IDD*

These examples are not listed in any order however they are among the most seen

- Down Syndrome
- Inborn Errors Of Metabolism
- Fragile X Syndrome
- Fetal Alcohol Syndrome Disorder
- Cerebral palsy
- Autism Spectrum Disorder

**Slide 18:** Application of the ICF Model

*The facilitator(s) will explain how to apply the ICF model to a patient who has cerebral palsy with contractures*

- An example of how to apply this model, think of a patient with cerebral palsy, keeping in that no two patients with CP present the same way or will have the same needs, looking specifically at body functions and structures we focus on joint function, the impairment being contractures. What activity limitations can this lead to? Difficulty walking for example. Which can lead to participation limitation by restricting access to education and community life.
- Now looking at environmental factors: what aspects of the world around this individual are either facilitators or barriers, think of need for personal assistance- not mentioned here but if this patient were wheelchair dependent are there ramps at home or in school. And then personal factors looking at gender, age, education level, motivation.

**Slide 19:** ICF model example of panic disorder

*The facilitator will describe how to apply the ICF model to an impairment of the mind*

- In a person with panic disorder the impair could be a problem of emotions which can make it difficult for them to handle stress and other psychological demands. This can then lead to restricted social relationships.
- Environmental factors that can influence this person’s experience or quality of life could be barriers such as negative attitudes and lack of support systems.
- Personal factors can include the person’s age and gender

**Slide 20:** Group activity application of the ICF model

*The facilitator(s) will engage the participants in a “Think-Pair-Share” activity applying the ICF model to the common causes of IDD recently discussed*

- Now, going back to the ICF model and considering these common causes of IDD, select one of the common causes of IDD we just discussed for example Down Syndrome or Cerebral palsy and using the ICF model identify one potential impairment (body function/structure) and one environmental barrier that that individual might face. Please take 2 minutes to think about this prompt.
- Then, turn to a neighbor and take 3 minutes to share your choices. Discuss how changing an environmental factor (from a barrier to a facilitator) could directly improve that person's participation in their community.

*After this, the facilitator(s) will ask the audience for 1-2 volunteers* to share their scenarios with the larger group, illustrating how disability is an interaction rather than just a medical state.

**Slide 21:** Defining medical complexity

*The facilitator will define medical complexity and what determines it*

- Not all patients with disabilities have complex medical needs and the opposite is true.
- There are many definitions for this term, none of which are standardized.
- The terms complex care or complex medical needs describe a heterogeneous population with a wide spectrum of health challenges
- Overall, some of the commonalities that we see among these patients are that they tend to be affected by multisystem diseases requiring specialty care. They often have recurring presentations to care and frequent reliance on medical technology. Because of their multiple healthcare needs they are Highly dependent on care coordination with needs that may change over time, and are at increased risk of fragmented care, sentinel events and difficulties accessing appropriate healthcare resources due to the number of different medical specialists involved in their care.
- Unfortunately, these experience considerable health disparities throughout their lifetime, including decreased life expectancy and greater comorbidities

**Slide 22:** Common Comorbidities

*The facilitator will describe common comorbidities that affect patients that are considered medically complex*

- Patients with medical complexity are often affected by multisystem disorders. Progression or complications from these disorders, or side effects from medications used to treat some of them, can lead to comorbidities such as:
  - Gastroesophageal reflux disease
  - Constipation
  - Musculoskeletal disorders- scoliosis, contractures, spasticity
  - Seizure disorder
  - Mental health/behavioral disorders
  - vision/dental/hearing impairment

**Slide 23:** Approach to the patient

*The facilitator will describe how to approach patients with IDD and medical complexities*

- When caring for patients with IDD and complex medical needs, we still use the same general framework that we use for all patients, ensuring to ask a thorough medical history. Still, we must consider other challenges and diagnoses that this population may also face.
- For example, when thinking about past medical or current medical problems, we must keep in mind that:
- There is a higher prevalence of mental health conditions, physical health conditions, and co-morbidities than in the general population, and they are more likely to develop chronic conditions. For example, COPD, asthma, diabetes, and heart failure all have a higher prevalence in those with IDD than in the general population. Therefore, how can we prevent these from developing or worsening if already present?
- Learning about the birth history/Development is important and can help learn more about the person’s condition
- A good medication reconciliation is especially important for this patient due to the risk of polypharmacy and potential side effects
- There are special considerations to keep in mind for social history which we will discuss in the upcoming slides
- Review of systems is an opportunity to determine if there are new problems and prevent further complications

**Slide 24:** Approach to the patient Cont’d

*The facilitator will describe how to approach patients with IDD and medical complexities*

- To understand our patients' needs, we must adopt a comprehensive approach that includes a baseline assessment of their functioning and the etiology of their IDD.
- The American Academy of Pediatrics has been striving for a seamless transition of care for children entering adulthood, especially those with IDD. Guidelines recommend starting this transition at 12 years of age.
- The etiology of the IDD is important to verify, as it often guides further workup and health-care services that should be offered.
- A baseline assessment of intellectual and physical functioning is recommended to establish a baseline and help gauge future acute or chronic changes that can inform care planning.
- It's important to note who the specialists they have been seeing are or if they have been lost to follow-up during the transition to adult care.

**Slide 25:** Social history

*The facilitator will describe special considerations in the social history for patients with IDD and medical complexities*

- Special considerations to keep in mind when approaching the social history for patients with IDD include the transition of adolescent patients to adult care, which includes having conversations about guardianship early, before the age of 18
- Obtain a sexual history and don’t make assumptions; allow privacy to patients from their caregivers
- Considering their home environments, are they still in school, and if they have graduated, are they attending an adult day care? If not, who is caring for them at home, and what level of care do they require? Do they need services such as home health aid services?
- For older patients, who will care for them in the future as their parents and siblings also continue to age?
- Last, is there any concern for abuse, especially in a non-verbal patient. This would require a high index of suspicion and must be reported to protective services.

**Slide 26:** Physical exam

*The facilitator will discuss special considerations when performing the physical exam on a patient with disability.*

- There are many considerations to keep in mind when examining patients with disabilities, starting with accessibility. The clinic/room must be able to accommodate these patients, for example, by having wide doors and large rooms for wheelchairs/stretchers, wheelchair accessible scales, adjustable tables etc.
- You must have behavioral and emotional sensitivity, be patient and flexible, and consider desensitization techniques to help with anxiety, such as showing the patient equipment like the stethoscope and allowing them to touch it.
- Involve caregivers when needed; they know their relative best and can assist with some of these calming techniques and transfers to and from the examining table.
- When examining the patient, pay attention to their facial expressions, especially for non-verbal patients, keeping in mind that abnormalities in vital signs could be signs of anxiety or pain and should be explored further.

In the next couple of slides, I would like to discuss a case study with you, where many of these considerations come into play. It would be appreciated if you could all participate and share your thoughts/ask questions. There are no wrong answers; this is just a discussion.

**Slide 27:** Case Study: Meeting Kira

*The facilitator(s) introduce Kira’s case*

- Kira is a 21-year-old young woman with a history of Trisomy 21, mild intellectual disability, history of global developmental delay, Hyperthyroidism, Asthma, and Atrioventricular Septal Defect post surgical repair, who presents to the Med Peds clinic for a routine annual exam with her mother, after they moved from North Carolina to New Jersey.
- Her mother states that unfortunately they had lost their insurance, so Kira has not been to the doctor for a few years. Her mother is very concerned about Kira's health and wants to make sure she gets re-established with a healthcare team.
- Kira, a shy, yet sociable 21-year-old, shares that she feels well today, but as a dedicated photography student, she is frustrated that her blurry vision makes it difficult to follow her teacher's notes on the board.
- Kira also expresses that she would like to be more active and involved in her care during medical appointments as she navigates her transition to a new adult healthcare team

**Slide 28:** Case Study additional information

*The facilitator will summarize what we know about Kira and add additional information regarding her current health status*

- In terms of her past medical history Kira used to see a cardiologist and endocrinologist regularly for her history of AVSD and hyperthyroidism, however, was lost to follow up when their insurance was interrupted.
- Her asthma is well controlled. Only has albuterol and uses it once every few months. Has never been to a pulmonologist. There are no issues with her menstrual cycles, they are not painful. Her only medication currently is levothyroxine 75mcg which she takes daily however has not had lab work recently to adjust the dose.
- Her Mother is her primary caretaker. She helps her with her medications, but she can do many of her activities of daily living independently. She lives at home with her mother who is now aging and has complications from her own medical conditions and an older sister. She is not sexually active.
- She has mild IDD and enjoys working at an animal shelter. She sometimes attends an adult Down Syndrome Day Program where she is taking some classes like photography.
- Mother reports that she and her family are generally very happy, but she often worries about Kira and what the future will be like.
- Can briefly mention the review of systems (ROS); list the vitals (blood pressure is elevated, BMI is in the obesity category) and she has a murmur on heart exam.

**Slide 29:** Case Discussion

*The facilitator(s) will guide a discussion regarding important things to consider for Kira’s case*

*The facilitator(s) should ask for volunteers to share their thoughts*

- Questions to guide conversation: What are your first thoughts when meeting Kira? *Allow 5 seconds for a volunteer; if not, pick someone from the group.*
- Would Kira be considered to be someone with complex medical needs?
- How would you approach and coordinate the care of this patient?

*Facilitator to continue the discussion by stating the following*

- Kira has complex needs that cannot all be addressed in this visit, with multiple systems affected in part in relation to her trisomy 21.
- Some of the more urgent needs include setting up referrals and appointments with her cardiologist and obtaining lab results, keeping in mind her risk factors from the perspective of a patient with trisomy 21 and some of the risk factors identified in her medical history.
  - *For residents, ask: What kinds of labs would you order for her? Thyroid function tests, lipid panel, hba1c, hepatic function panel, CBC; What other referrals would you consider or tests: sleep study, ophthalmology*
- Blurry vision is important to address in patients with trisomy 21 because 60-80% of individuals with DS have vision problems (cataracts, strabismus, glaucoma, refractive errors). They should undergo regular vision screenings at all health supervision visits or be evaluated by an ophthalmologist specializing in disabilities every 2 years.
- In a patient with Down syndrome who is snoring, consider obstructive sleep apnea (OSA). Patients with trisomy 21 frequently have OSA (50-79%). This can contribute to cardiovascular problems as well, and she has a history of CHD.
- There are many things that we will need to consider when caring for Kira; however, these considerations will need to be addressed over multiple visits as she re-establishes her healthcare. During this visit, we address the most urgent issues first and then tackle the rest of the long-term problems/preventative health concerns in subsequent visits.

**Slide 30** Untangling the Complexity: The First Visit

*The facilitator(s) will continue to read Kira’s case*

- We decided to deal with her more urgent concerns first We decided to deal with her more urgent concerns first.
- For her cardiac history and new murmur we ordered an echo and refer her to a cardiologist
- For her thyroid disorder we order thyroid function tests and refer her to endocrinology
- For her snoring a sleep study was ordered to asses for obstructive sleep apnea
- And for her new onset of blurry vision a referral to ophthalmology was placed
- You schedule a follow-up visit for her in 3 months.

**Slide 31:** How can the ICF model be applied in this case?

*The facilitator(s) will provide an example of how the components of the ICF model can be applied to Kira’s case and care*

- Looking at the medical and social models of disability, Kira’s disability is not just her Trisomy 21 (Medical Model), but also her loss of insurance and her recent move which created environmental barriers to her care (Social Model).
- In this case, her health conditions include her trisomy 21 with mild intellectual disability
- As a result of her trisomy 21, the body functions and structures affected include her vision, heart and mind
- Activities & Participation: Her participation is directly impacted by her vision impairment, which makes it "hard to see what the teacher is writing" in her photography class
  - Her involvement in an adult daycare center is another key area of social participation
- Environmental Factors:
  - Barriers: Loss of health insurance, recent relocation to a new state
  - Facilitators: A supportive family (sister and mother)
- Personal Factors: She is a 21-year-old female

**Slide 32** Case #1 continued

*The facilitator(s) will continue to read Kira’s case and lead further discussion regarding her care*

- Kira returns to the clinic with her 25-year-old sister. You review her labs and imaging studies, which are essentially within normal limits, and you decide not to make changes to her Levothyroxine
- She is using CPAP at night and has established care with cardiology and endocrinology.
- Upon further questioning it is revealed that Kira's mom has been hospitalized for the second time this month and is currently admitted to the hospital.

*Facilitator to guide discussion, asking the following question: How does this affect Kira's care and what resources do we want to make available for her and her sister? Give the audience a few seconds to participate. If there are no answers or minimal answers, the facilitator can say:*

- We need to consider Kira's care if her mother were no longer able to care for her. This is something that we see more and more often as the lifespan of patients with IDD/complex medical needs continues to increase.
- You have to consider who Kira's legal guardians are. Is her sister one of them? And if not starting that process. Where would Kira live if her mother could no longer care for her, and who would bring her to appointments and take care of her healthcare needs?

**Slide 33** Guardianship and Supported Decision Making (SDM)

*The facilitator(s) explain what guardianship is and discuss less restrictive alternatives*

- By definition, a guardian is a person or agency that is appointed by a court to act on behalf of an individual.
- It is important to determine in the outpatient setting, usually around the time of transitioning to adulthood, so that by age 18, it has been defined. It's especially important if capacity/competence is in question, in case they have a medical emergency, so the care team will know who to contact.
- Guardianship can be general or limited. For example, it could be limited only to decisions involving education, finances, and medical.
- It should be a last resort because it takes away autonomy. This is a legal process, and once it's been established by the court, only the court can modify or change it.
- Less restrictive alternatives include:
  - A Power of Attorney (POA) which is a revocable alternative for specific decision-making
  - Supported Decision Making (SDM): which is a model where the individual retains their legal rights and makes their own choices with the help of a trusted support team. SDM is now recognized in various forms across many states as a way to preserve autonomy while ensuring safety.
- Other services that can help in this process include the Bureau of Guardianship Services at the Department of Human Services, and some families will also enlist the help of an attorney.
- When a transitioning patient is establishing care in your practice or you are seeing a new patient with IDD, you need proper documentation of guardianship.

**Slide 34:** The ICF Model and Supported Decision Making

*The facilitator(s) will explain how supported decision making can act as an environmental facilitator in a patient’s care*

- Supported decision making transitions the focus from the medical condition to the totality of the situation
- In the ICF model, an SDM support team can act as a critical Environmental Facilitator. By providing the right support (Environment), we can mitigate Activity Limitations and prevent Participation Restrictions, allowing individuals to direct their own care and life goals
- In the Kira’s case, using SDM empowers patients like Kira to be active and involved in her medical appointments

**Slide 35** Case #1 continued

*The facilitator(s) will continue to read Kira’s case*

- After a month, Kira returns to clinic with her mother and sister. Her mother is much more stable. They are currently working on obtaining shared guardianship over Kira's care for her sister, and Kira starts at the adult daycare center next week.
- You plan a follow up visit in 3 months where you will discuss preventative care.

**Slide 36** Health Disparities

*The facilitator(s) will introduce health disparities in patients with IDD*

keeping Kira's case in mind and what we've discussed so far. What are some of the health disparities we see in patients with IDD?

- To bridge the gaps in care for this patient population, we must understand and address the existing health disparities. Some of those include limited access to care, higher prevalence of chronic disease, health equity gaps, and barriers to preventative care, some of which we have already discussed.
- Limited access to care can be due to personal versus external or environmental factors (consider the ICF model). Such issues include mobility issues, communication difficulties, and a lack of proper accommodation
- Higher prevalence of chronic diseases, including obesity, diabetes, and cardiovascular disease, which can be due to a sedentary lifestyle, medication side effects, and limited access to nutritious foods- keep in mind the intersection with other social determinants of health.
- Health equity gaps encompass factors such as health literacy and social determinants of health. Lower health literacy can make it more challenging to manage chronic diseases.
- People with IDD are disproportionately affected by poverty, social isolation, and a lack of educational opportunities.
- These patients often require assistance with activities of daily living, and you have to consider caregiver burnout, which can affect their ability to provide proper care and lead to suboptimal management of health conditions.
- Lastly, barriers to preventive care may involve health programs that do not consider the unique needs of people with IDD. This can then lead to lower participation rates in activities like exercise or health education.
- To improve the health and well-being of individuals with IDD, it is crucial to understand the underlying causes of these disparities and implement strategies to reduce them.

**Slide 37** Health Promotion

*The facilitator(s) discuss how to promote health in patients with IDD*

How do we promote health in this patient population?

- Improving Access to Healthcare at the Provider, Institutional, and Systemic Levels. We start small. As physicians, we need to familiarize ourselves and gain confidence in caring for these patients.
  - We need to train Healthcare Providers as early as possible:
  - This includes focusing on communication strategies, patient-centered care, and recognizing common health issues for this population. (This could include learning about signs and symptoms of chronic conditions, as well as how to modify treatments or tests to meet the needs of patients with disabilities.) It could also involve obtaining knowledge of the health care system in which the provider works
  - Increase Access to Primary Care:
    - This includes ensuring that individuals with IDD have access to a primary care provider who is familiar with their specific needs, which can facilitate early diagnosis and effective long-term health management.
- Early Detection and Preventive Screenings, and promoting healthy lifestyles
  - Develop Regular Health Screenings that are adapted for individuals with IDD. For example, conducting age-appropriate screenings for heart disease, diabetes, and cancer can help catch problems early before they develop into chronic conditions.
  - Adopt universal design principles in healthcare settings, with the idea of making things more accessible for individuals with IDD. This may involve providing accessible information, making physical accommodations, and offering assistance with appointments.
  - Encourage community-based programs that are tailored to the individual's cognitive abilities and physical limitations.
- Addressing mental health care – ensuring that the proper mental health screening is being done at preventive visits and that they are connected with mental health professionals if needed
- Improving health literacy – recognizing the various levels of health literacy among patients and ensuring that information is delivered to them at their appropriate level to ensure understanding and participation in their health care.

**Slide 38** Preventative Care

*The facilitator(s) will describe the various preventative screenings that should be considered for our patients.*

- In general, according to the WHO, primary care can meet 80-90% of a person's health needs over their lifetime, and we want to ensure that this remains true for our patients with IDD.
- Preventative health includes reviewing immunization history, screening for hypertension at every visit
  - Screening for hyperlipidemia and diabetes
  - Age-appropriate cancer screening
  - Depression screening during an annual visit or when there is a concern
  - Additional screening based on risk factors such as high-risk behaviors, or smoking, for example
  - Osteoporosis screening, keeping in mind that in specific patient populations, you will need to screen earlier than for the general population, think of a patient with cerebral palsy who has been wheelchair dependent most of their life
  - Advance care planning

**Slide 39** Summary slide, health promotion requires a multidisciplinary approach

*The facilitator(s) will summarize the contents of the module*

- The goal is to have a medical home for patients with IDD that involves coordinated and comprehensive care.
- To summarize:
  - Disability refers to physical or mental conditions that may limit a person’s ability to perform everyday activities or engage with others.
  - Individuals with IDD are often medically complex, requiring care across multiple systems and specialties.
  - The ICF model can serve as a framework for thorough and collaborative patient care
  - Ultimately, effective care for patients with IDD involves a comprehensive, long-term, and preventative approach to reduce health disparities and improve outcomes.

**Slide 40 and 41** References

1. Bobbette N, Ouellette-Kuntz H, Tranmer J, Lysaght R, Ufholz LA, Donnelly C. Adults with intellectual and developmental disabilities and interprofessional, team-based primary health care: a scoping review. JBI Evid Synth. 2020;18(7):1470-1514. doi:10.11124/JBISRIR-D-19-00200
2. Krahn GL, Fox MH. Health disparities of adults with intellectual disabilities: what do we know? What do we do? J Appl Res Intellect Disabil. 2014;27(5):431-446. doi:10.1111/jar.12067
3. Teed M, Bekx A, Paul M, Younker L. Health care transition for children with medical complexity: challenges and lessons learned. J Pediatr Nurs. 2021;61:275-279. doi:10.1016/j.pedn.2021.06.017
4. Centers for Disease Control and Prevention. Disability and health overview. Centers for Disease Control and Prevention. Accessed October 13, 2025. https://www.cdc.gov/disability-and-health/about/index.html
5. Centers for Disease Control and Prevention. Disability and health overview [image]. Accessed June 11, 2026. https://www.cdc.gov/disability-and-health/about/index.html
6. Centers for Disease Control and Prevention. ICD-10 Overview and Update. Centers for Disease Control and Prevention; 2020. Accessed October 13, 2025. https://www.cdc.gov/nchs/data/icd/icfoverview_finalforwho10sept.pdf
7. American Psychological Association. Disability models. Psychology Teacher Network. Published 2021. Accessed June 11, 2026. https://www.apa.org/ed/precollege/psychology-teacher-network/introductory-psychology/disability-models
8. Centers for Disease Control and Prevention. ICF overview [image]. Accessed February 26, 2025. https://www.cdc.gov/nchs/data/icd/icfoverview_finalforwho10sept.pdf
9. Centers for Disease Control and Prevention. Disability Impacts All of Us (Infographic). CDC; 2020. Accessed October 13, 2025. https://www.cdc.gov/disability-and-health/media/pdfs/disability-impacts-all-of-us-infographic.pdf
10. Moeschler JB, Shevell M. Comprehensive evaluation of the child with intellectual disability or global developmental delays. Pediatrics. 2014;134(3):e903-e918. doi:10.1542/peds.2014-1839
11. Teed M, Bekx A, Paul M, et al. Health care transition for children with medical complexity: challenges and lessons learned. *J Pediatr Nurs*. 2021;61:275-279. doi:10.1016/j.pedn.2021.06.017
12. Hogan AK, et al. A tertiary care-based complex care program: improving care for children with medical complexity. *Med Care.* 2020;58(11):958-962. doi:10.1097/MLR.0000000000001388
13. Daaleman TP. Special population: adults with intellectual and developmental disabilities. In: Daaleman T, Helton M, eds. *Chronic Illness Care.* Springer; 2018. doi:10.1007/978-3-319-71812-5_26
14. Wellness_photos. Child at Doctor - Kid at Doctor's Office [image]. Accessed June 11, 2026. Licensed under CC BY-SA 2.0.
15. New Jersey Department of Human Services. Guardianship. Accessed October 13, 2025. <https://www.nj.gov/humanservices/ddd/individuals/guardianship/>
16. Krahn GL, Fox MH. Health disparities of adults with intellectual disabilities: what do we know? What do we do? *J Appl Res Intellect Disabil.* 2014;27(5):431-446. doi:10.1111/jar.12067
17. Sonderlund AL, Baygi F, Soendergaard J, Thilsing T. Advancing health equity for populations with intellectual disabilities: a systematic review of facilitators and barriers to the implementation of health checks and screening. *Public Health.* 2024;229:29-36. doi:10.1016/j.puhe.2024.02.002
18. Bull MJ, Trotter T, Santoro SL, Christensen C, Grout RW. Health supervision for children and adolescents with down syndrome. *Pediatrics.* 2022;149(5):e2022057010. doi:10.1542/peds.2022-057010
19. Laura4Smith. Doctor Visit [image]. Accessed June 11, 2026. Licensed under CC BY-NC-ND 2.0.

**Slide 42** Questions

*The facilitator(s) will leave room for any questions. Remind the participants to complete the post-session assessment.*
