# Supplementary material for: Bridging the Gap: Improving Health Outcomes for Adults With Intellectual and Developmental Disabilities
Source: MedEdPORTAL. 2026 Aug 21;22:11630. doi: 10.15766/mep_2374-8265.11630 (PMC13493612; doi:10.15766/mep_2374-8265.11630)
Supplement: Supplementary file 1 [file mep_2374-8265.11630-s001.zip › A. Presession Evaluation.docx]

**This is the pre workshop survey. This document should be distributed to the participants before the workshop.**

Q1 Please create a 5-digit PIN using the FIRST initial of your LAST NAME and the last 4 digits of your phone number. (ie: P1914 you will also be putting this on your post survey to link them)

________________________________________________________________

Q2 Educational or Professional Role

- College or Post-bacc Student
- Medical Student
- Medical Resident or Fellow
- Academic Faculty (MD, DO, NP, PA, PhD, Other)
- Physician in Non-Academic Practice (MD, DO, NP, PA, Other)
- Other

Q3 What is your race/ethnicity (choose all that apply)

- White
- Black or African American
- Hispanic, Latino, or Spanish Origin
- Middle Eastern or North African
- American Indian or Alaska Native
- Asian
- Other

Q4 Sexual Orientation

- Straight or heterosexual
- Gay or lesbian
- Bisexual
- Other
- Prefer not to say

Q5 How do you self-identify? Please check all that apply

- Gender non-binary, gender non-conforming, genderqueer
- Man
- Woman
- Transgender
- Prefer not to say

Q6 In which state or commonwealth is your academic institution located?

________________________________________________________________

**End of Block: Demographics**

**Start of Block: Care of the complex patient**

Q7 Please rate how much you agree with the following statements

|  | Strongly disagree (1) | Somewhat disagree (2) | Neither agree nor disagree (3) | Somewhat agree (4) | Strongly agree (5) |
| --- | --- | --- | --- | --- | --- |
| I can define what it means to have a disability |  |  |  |  |  |
| I am familiar with the ICF model and I feel comfortable applying it in the clinical setting |  |  |  |  |  |
| I know the difference between developmental delay, developmental disability and intellectual disability |  |  |  |  |  |
| I know what entails medical complexity |  |  |  |  |  |
| I received sufficient education and training in the care of patients with intellectual and developmental disabilities with complex medical needs |  |  |  |  |  |
| I feel confident caring for patients with developmental/intellectual disability and complex medical needs |  |  |  |  |  |
| I am familiar with some of the health disparities faced by patients with IDD |  |  |  |  |  |

**End of Block: Care of the complex patient**
